# A new species of *Acerentulus* Berlese, 1908 (Protura, Acerentomata, Acerentomidae) from Bulgaria with a revised key to the *confinis* group

**DOI:** 10.3897/zookeys.876.36743

**Published:** 2019-09-18

**Authors:** Julia Shrubovych, Dilian G. Georgiev, Cristina Fiera

**Affiliations:** 1 Institute of Systematics and Evolution of Animals, Polish Academy of Science, Sławkowska 17, Pl 31-016 Krakow, Poland Institute of Systematics and Evolution of Animals, Polish Academy of Science Krakow Poland; 2 State Museum of Natural History, Ukrainian Academy of Sciences, Teatral’na St. 18, UA 79008 Lviv, Ukraine Ukrainian Academy of Sciences Lviv Ukraine; 3 Institute of Soil Biology, Biology Centre, Czech Academy of Sciences, Na Sádkách 7, 370 05 České Budějovice, Czech Republic Institute of Soil Biology, Biology Centre, Czech Academy of Sciences České Budějovice Czech Republic; 4 University of Plovdiv, Tzar Assen Str. 24, BG-4000 Plovdiv, Bulgaria University of Plovdiv Plovdiv Bulgaria; 5 Institute of Biology Bucharest of Romanian Academy, 296 Splaiul Independenţei, P.O. Box 56-53, 060031, Bucharest, Romania Institute of Biology Bucharest of Romanian Academy Bucharest Romania

**Keywords:** Chaetotaxy, Holarctic, identification key, porotaxy, proturans.

## Abstract

A new species, *Acerentulus bulgaricus***sp. nov**., belonging to the *confinis* group, is described from Bulgaria. This species is characterized by long foretarsal sensilla *a* and *b*, the posterior position of foretarsal seta δ*4*, the presence of seta *P1a* on abdominal tergites II–VII and seta *P3a* on abdominal tergite VII, possession of eight anterior setae on abdominal tergite VII and composed *spsm* pores on sternite VI. The new species differs from all members of the *confinis* group in possessing *P1a* setae on tergites II–VII. Otherwise it is similar in body chaetotaxy and porotaxy to three species of the *cunhai* group, *A. proximus*, *A. correzeanus* and *A. tuxeni*. The identification key to 22 *Acerentulus* species belonging to *confinis* group is revised.

## Introduction

The proturan genus *Acerentulus* Berlese, 1908 is widely distributed over the Holarctic, reaching Southern America, Australia and New Zealand. According to the Catalogue of the World Protura (Szeptycki 2007), the genus comprises 40 species and 2 subspecies. *Acerentulus rapoporti* Condé, 1963, which was noted by Szeptycki (2007) as “species incertae sedis”, was recently synonymized with *Andinentulus ebbei* (Tuxen, 1984) (Shrubovych et al. 2014a). Several species, noted as “species inquirendae” in Szeptycki (2007), are not placeable; type materials were lost for *Acerentulus americanus* Hilton, 1943 and *A. shensiensis* Chou & Yang, 1964, and *A. aubertoti* Condé, 1944 was described from a prelarva (see Tuxen 1955, Szeptycki 2007). Since Szeptycki (2007) additional species have been described, bringing the current total to 48 species (Wu and Yin 2007, Shrubovych et al. 2012, 2014b, Galli and Capurro 2013, Galli et al. 2017). Distributions and taxonomic differentiation between the 21 species within the *confinis* group was discussed previously (Shrubovych et al. 2012). The present paper contains a description of a new *Acerentulus* species from Bulgaria, which belongs to the *confinis* group. Only three species have been recorded from Bulgaria till now: *Acerentulus confinis* (Berlese, 1908), *A. gisini* Condé, 1952 and *A. traegardhi* Ionesco, 1937 (Szeptycki 2007) contrary to well-studied neighboring countries: 11 and 8 *Acerentulus* species were recorded in Serbia and in Romania respectively (Blesić and Mitrovski-Bogdanović 2012, Shrubovych and Fiera 2016). An identification key to the *confinis* group of species worldwide is updated and reorganized according to morphological characters.

## Material and methods

Protura specimens, collected in Bulgaria from 2015 to 2018, were extracted from soil samples with Berlese-Tullgren funnels into 95% ethanol. All specimens were mounted on glass slides in Faure’s medium (Dunger and Fiedler 1989). Additional material was analyzed in the collection of J. Rusek, deposited in the Institute of Soil Biology, Biology Centre of the Czech Academy of Sciences (**ISB**).

The holotype and other materials of D. Georgiev and C. Fiera are deposited in the collection of the Institute of Systematics and Evolution of Animals, Krakow, Poland (**ISEA**). One female paratype (ISB A-791.1) and materials of J. Rusek are deposited in the collection of the Institute of Soil Biology, Biology Centre, Czech Academy of Sciences. One female paratype (SMNH 90.1) is deposited in the collection of the State Museum of Natural History, Lviv, Ukraine (**SMNH**).

The morphological characteristics of the genus are given in Nosek (1973), Imadaté (1988), Szeptycki (1991), and Galli et al. (2018). Some new details concerning development and variability of chaetotaxy and porotaxy are added. Information on the taxonomy of *Acerentulus* species was taken from original descriptions or redescriptions of type materials in Nosek (1973). For description of morphological characters, the terminology used by Rusek et al. (2012), Szeptycki (1991) and Shrubovych (2014) was followed for this study. Abbreviations used in the description are as follows: Abd. = abdominal segments, Th. = thoracic segments, *al* = anterolateral pore, *sl* = sublateral pore, *psl* = posterosublateral pore, *psm* = posterosubmedial pore, *spm* = sternal posteromedial pore, *spsm* = sternal posterosubmedial pore.

## Results

### Acerentulus bulgaricus

Taxon classificationAnimaliaProturaAcerentomidae

Shrubovych sp. nov.

92C9B965-8D70-5CF0-B58F-CB3E24D8C4C1

http://zoobank.org/28AA95C1-B361-4A22-9D4D-AE714690241D

**Figure 1.**
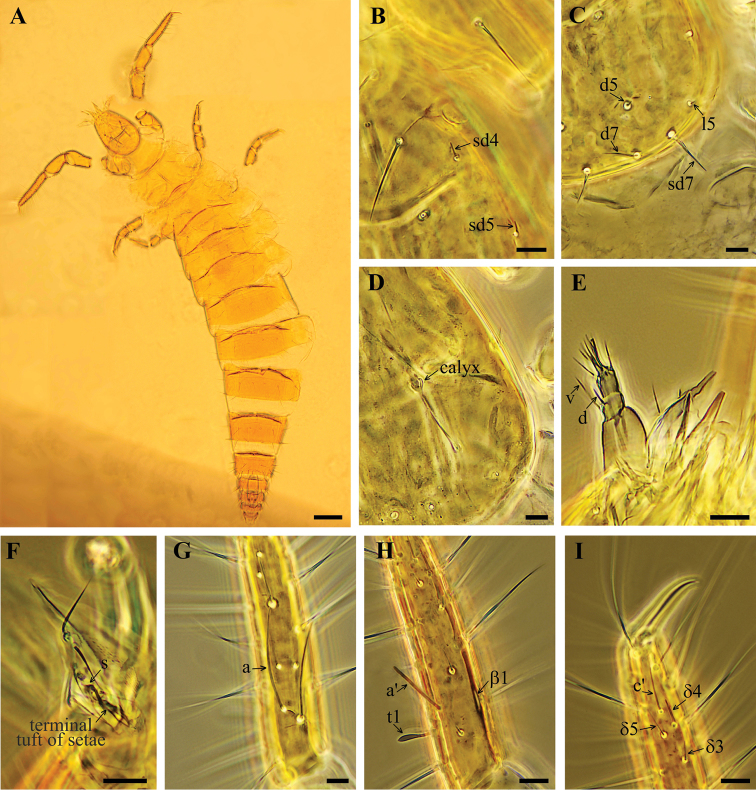
*Acerentulus bulgaricus* sp. nov.: Holotype **A** habitus **B** lateral part of head **C** hind part of head **D** maxillary gland **E** maxillary palpus **F** labial palpus **G–I** exterior view of foretarsus. Scale bars: 100 µm (**A**), 10 µm (**B–I**).

**Figure 2.**
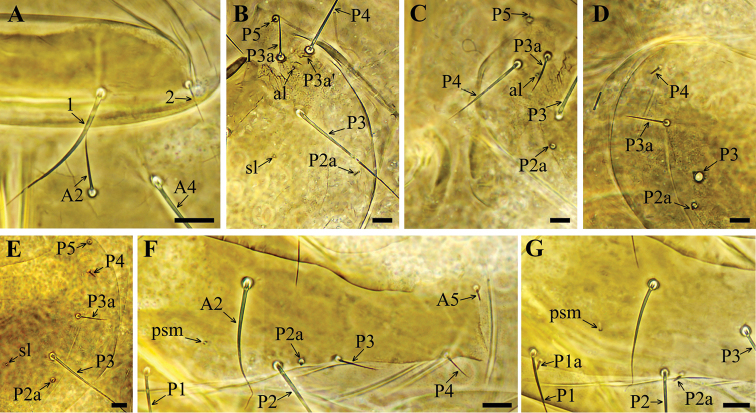
*Acerentulus bulgaricus* sp. nov. **A** part of pronotum **B** part of mesonotum **C** part of mesonotum **D** part of metanotum **E** part of metanotum **F** part of tergite I **G** part of tergite II. Figures **B, D** – paratype SMNH 90.1 **A, C, E, F, G** – holotype. Scale bars: 10 µm.

**Figure 3.**
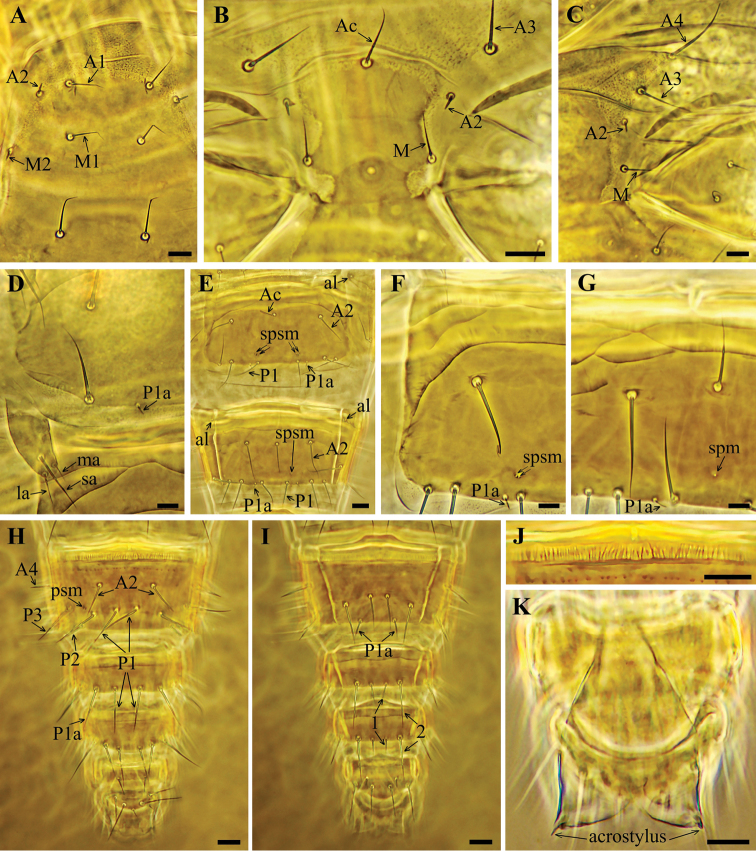
*Acerentulus bulgaricus* sp. nov. **A** prosternum **B** mesosternum **C** metasternum **D** part of sternite I **E** sternites VI–VII **F** part of sternite V **G** part of sternite VII **H** tergites VIII–XII **I** sternites VIII–XII **J** striate band on tergite VIII **K** female squama genitalis. Figures **B, D** – paratype ISB A-791.1 **A, C, E–K** – holotype. Scale bars: 10 µm.

**Figure 4.**
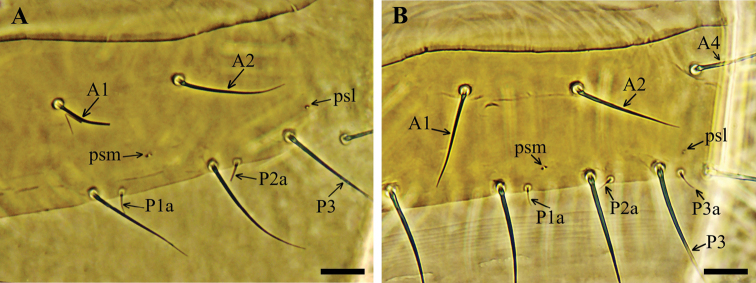
*Acerentulus bulgaricus* sp. nov.: maturus junior (Sarnena Gora. 6.VI.2017. coll. D. Georgiev). **A** part of tergite VI **B** part of tergite VII. Scale bars: 10 µm.

**Figure 5.**
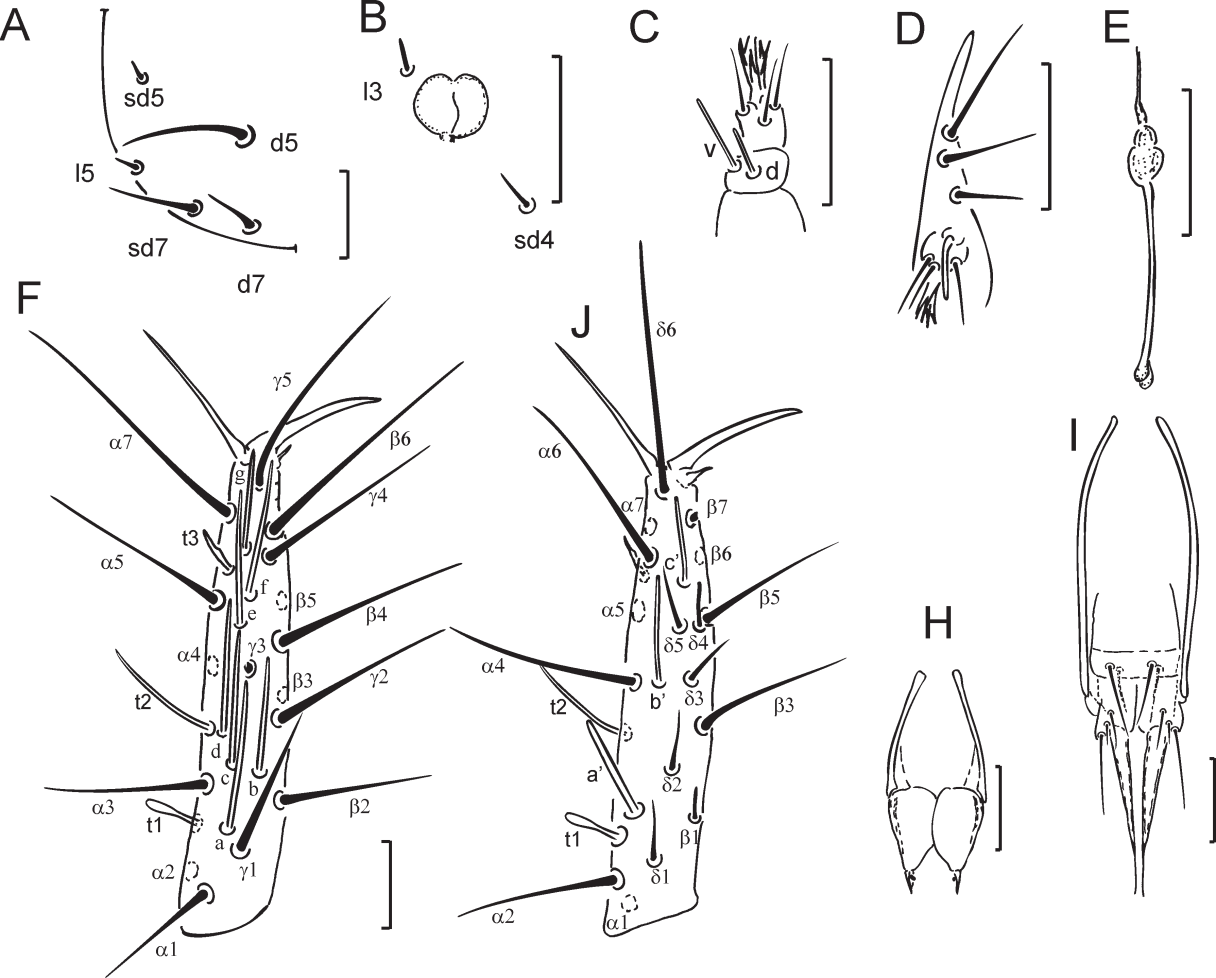
*Acerentulus bulgaricus* sp. nov.: **A** hind part of head **B** pseudoculus **C** maxillary palpus **D** labial palpus **E** maxillary gland **F** exterior view of foretarsus **G** interior view of foretarsus **H** female squama genitalis **I** male squama genitalis. Figure **H** – paratype SMNH 90.1 **A–G, I** – holotype. Scale bars: 20 µm.

**Figure 6.**
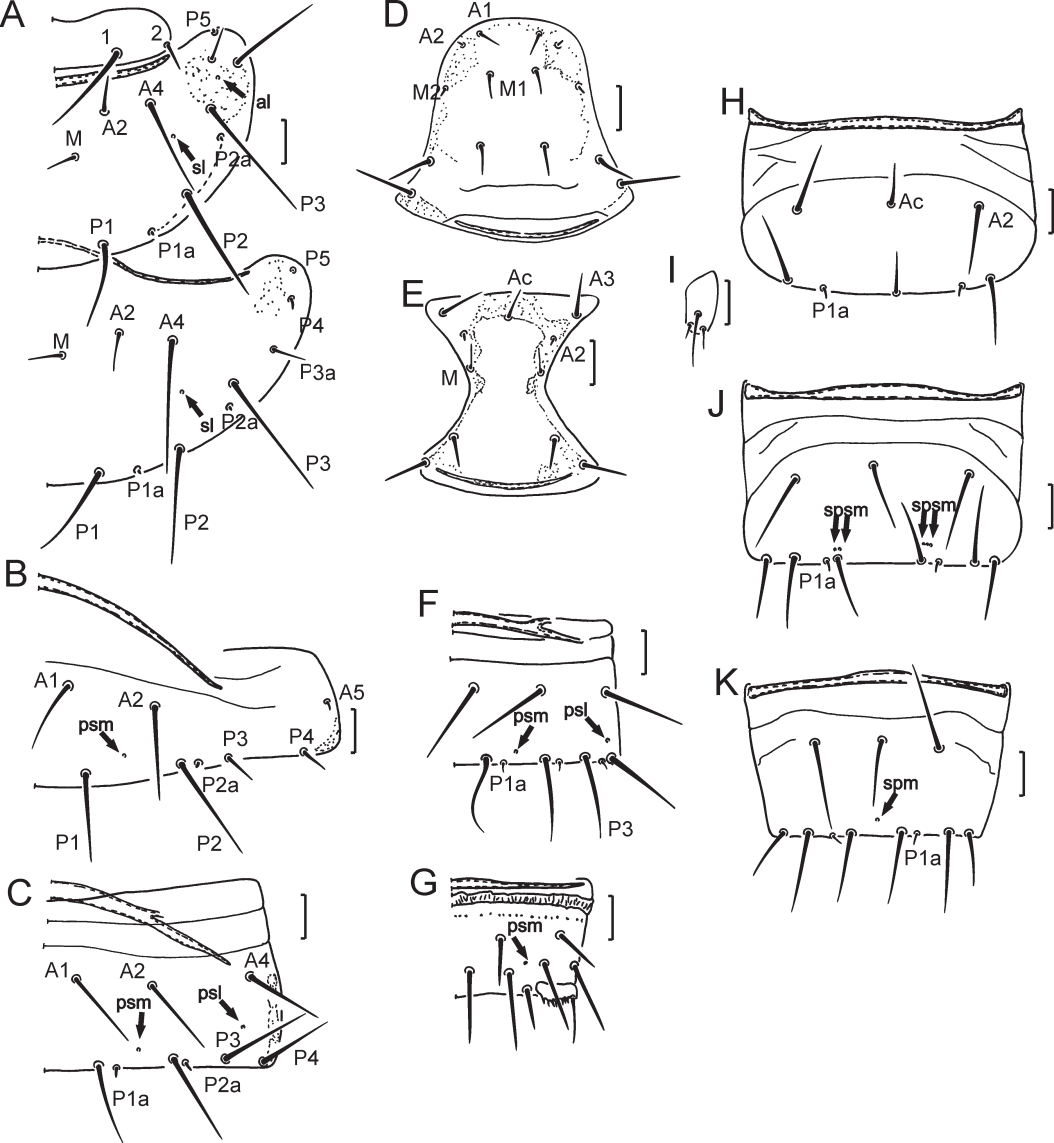
*Acerentulus bulgaricu*s sp. nov.: Holotype **A** part of pronotum and mesonotum **B** part of tergite I **C** part of tergite VI **D** prosternum **E** mesosternum **F** part of tergite VII **G** part of tergite VIII **H** sternite III **I** abdominal leg of sternite III **J** sternite VI **K** sternite VII. Arrows show pores. Scale bar: 20 μm.

Figs 1 , 2 , 3 , 4 , 5 , 6 ; Table 1

#### Material examined.

***Holotype***: male (ISEA 6649): Bulgaria, Black Sea coast, near Tsarevo, Popska River, moss, soil and detritus, 42°10'31.7"N, 27°50'21.3"E, 16 m elev., 26.VI.2017, coll. D. Georgiev. ***Paratypes***: 2 females (ISB A-791.1 and SMNH 90.1) same data as holotype. Other material: 10 females, 8 males, 1 preimago, 7 maturi juniores, 2 larvae II, 1 larva I, Bulgaria, St. Kirik and Yulita Monastery near Plovdiv, *Carpineto*-*Fagetum* forest, sample at decaying stump, 13.VI.1990, coll. J. Rusek; 4 females, 2 males, 1 preimago, 2 larvae II, Bulgaria, south foothills of Stara Planina, near Gurkovo town, mixed forest with *Robinia pseudoacacia* (L.) Gaerth., soil, 42°41'19.10"N, 24°45'09.08"E, 372 m elev., 30.VIII.2015, coll. C. Fiera; 2 females, Bulgaria, Sarnena Gora Mountains, near Kolena village, bank of stream, soil and detritus in roots of *Alnus glutinosa* (L.) Gaerth., 42°29'62"N, 25°41'28.61"E, 300 m elev., 15.VI.2017, coll. D. Georgiev; 1 maturus junior, 1 larva II, Bulgaria, Sarnena Gora Mountains, near Kolena village, *Pinus nigra* J. F. Arnold., soil and detritus, 42°24'03.1"N, 25°34'09.8"E, 296 m elev., 6.VI.2017, coll. D. Georgiev.

#### Diagnosis.

Setae *P1a* present on tergites II–VII, absent on tergite I; setae *P3a* present on tergite VII. Abdominal tergites VI–VII with eight anterior setae. Sternites I–III without pores, sternites IV–V with 1+1 *spsm* pores, sternite VI with composed *spsm* pores (2+2 or 2+3 pores placed adjacent to each other), sternite VII with a spm pore. Foretarsal sensilla *a*, *b* and *c* long, setae β*1* and δ*4* thick, stout and sensilliform, δ*4* situated proximally to the level of sensillum *c*’ base.

#### Description.

Habitus is shown on Figure 1A. Head setae *l3*, *sd4* and *sd5* short thickened sensilla, additional seta *d6* lacking (Figs 1B, C, 5A, B), length ratio of posterior setae *d7*:*sd7* as 1.0:1.4 (Fig. 5A). Pseudoculus circular, with indistinct posterior extension, PR = 15–17 (Fig. 5B). Sensilla of maxillary palps slender, differing in length, dorsal (*d*) sensillum shorter than ventral (*v*) (Figs 1E, 5C). Labial palps with four-branched tuft of apical setae and a slender sensillum (Figs 1F, 5D). Maxillary gland with rounded calyx, long and slender posterior filament and bilobed posterior dilation (Figs 1D, 5E), CF = 4.4–5.5.

Foretarsus with *t1* claviform, *t3* leaf-like and the same length as *t1* (Figs 1H, 5F, J). All other sensilla slender, except the broadened sensillum *a*’ (Figs 1H, 5J). Sensillum *a* long, reaching base of seta γ*3; b* and *c* long, extending past base of seta γ*3*, *b* slightly shorter than *c* (Figs 1G, 5F). Base of *d* close to *c*, near *t2* insertion; *a*’ situated distal to *t1* insertion (Figs 5F, J). Relative length of sensilla: (*t1 = t3) < (b’= c’) < a’< g < t2 < e < (c = d) < f < b < a.* Setae β*1* and δ*4* sensilliform and thickened, each 7 µm long (Figs 1H, I, 5J). Seta δ*4* situated on the level of δ*5*, proximal to *c*’ base (Figs 1I, 5J). Single pores situated near bases of sensilla *c* and *t3*. Claw long, without inner tooth, empodial appendage short. BS = 0.3, TR = 3.7–4.1, EU = 0.1.

Formula of chaetotaxy given in Table 1. Setae on nota strongly differing in length (Fig. 6A). Length ratio of pronotal setae *1*: *2* as 3.2: 1 (Figs 2A, 6A). Setae *P1a* and *P2a* on mesonotum and metanotum as small gemmate microchaetae, *P4* on metanotum sensilliform, short and thick (Figs 2B, C, D, E, 6A). Seta *P2a* situated close to *P3*. Length ratio of *P1*: *P2* on mesonotum as 1:1.2–1.4. Mesonotum with *sl* and *al* pores, metanotum with *sl* pores only (Figs 2B, E, 6A). Thoracic sterna without pores (Figs 3A, B, C, 6D, E). Setae *A2* on sterna and *M2* on prosternum short sensilliform and thickened (Figs 3A, 6D).

**Table 1.** Body chaetotaxy of *Acerentulus bulgaricus* sp. nov. Shrubovych.

	**Dorsal**		**Ventral**	
	**Setae**	**Formula**	**Setae**	**Formula**
Th. I	1, 2	4	A1, 2, M1, 2	4+4
			P1, 2, 3	6
Th. II	A2, 4, M	6	Ac, 2, 3, M	5+2
	P1, 1a, 2, 2a, 3, 3a, 4, 5	16	P1, 3	4
Th. III	A2, 4, M	6	Ac, 2, 3, 4, M	7+2
	P1, 1a, 2, 2a, 3, 3a, 4, 5	16	P1, 3	4
Abd. I	A1, 2, 5	6	Ac, 2	3
	P1, 2, 2a, 3, 4	10	P1, 1a	4
Abd. II–III	A1, 2, 5	6	Ac, 2	3
	P1, 1a, 2, 2a, 3, 4, 4a, 5	16	Pc, 1a, 2	5
Abd. IV–V	A1, 2, 5	6	Ac, 2	3
	P1, 1a, 2, 2a, 3, 4, 4a, 5	16	P1, 1a, 2, 3	8
Abd. VI	A1, 2, 4, 5	8	Ac, 2	3
	P1, 1a, 2, 2a, 3, 4, 4a, 5	16	P1, 1a, 2, 3	8
Abd. VII	A1, 2, 4, 5	8	Ac, 2	3
	P1, 1a, 2, 2a, 3, 3a, 4, 4a, 5	18	P1, 1a, 2, 3	8
Abd. VIII	A1, 4, 5	6	1, 2	4
	P1, 2, 2a, 3, 3a, 4, 4a, 5	16	1a	2
Abd. IX	1, 1a, 2, 2a, 3, 4	12	1, 2	4
Abd. X	1, 1a, 2, 2a, 3, 4	12	1, 2	4
Abd. XI	1, 3, 4	6	–	6
Abd. XII	–	9	–	6

Seta *P2a* on tergite I of same shape as *P1a* and *P2a* on nota, *P3* and *P4* short and setiform; *A5* a short thickened sensillum (Figs 2F, 6B). Accessory setae *P1a*, *P2a* and *P4a* on tergites II–VI short, sensilliform and thick, on tergite VII setae *P1a*, *P2a*, *P3a* and *P4a* thin and setiform (Figs 2G, 6C, F). Position of seta *P3* on tergites II–V anterior to other *P*-setae, on tergites I and VI–VII *P3* in the *P*-setae row (Figs 2F, G, 6B, C, F). Tergites II–VII each with a transverse connecting line in the anterior region (Fig. 6C, F). Pores *psm* on tergites I–VII, *al* on tergites II–VII, *psl* on tergites VI–VII (Figs 2F, G, 6B, C, F). Abdominal legs I with 4 setae, abdominal legs II and III with 3 setae: medial apical (ma), lateral apical (la) and subapical (sa) (Figs 3D, 6I). Accessory setae on sternites I–VI the same length (4 µm) and sensilliform as on tergites (Figs 3F, 6H, J). Accessory setae on sternite VII the same shape and length as on tergite VII (Figs 3G, 6K). Sternites II–III each with a connecting line anteriorly and with short lines in the anterolateral region (Fig. 6H); sternites IV–VI with two connecting lines, sternite VII with one connecting line (Figs 6J, K). Sternites I–III without pores. Sternites IV–V with 1+1 *spsm* pores (Fig. 3F), sternite VI with composed *spsm* pores (2+2 or 2+3) (Fig. 6J), sternite VII with single *spm* pore (Figs 3G, 6K).

Abdominal segment VIII with distinct striate band; tergite with a transverse row of small teeth and sternite with two rows of teeth (Figs 3J, 6G). Comb VIII with 10–12 small teeth (Fig. 6G). Pore *psm* without accompanying teeth. Posterior margin of sternite VIII and laterotergites smooth (Fig. 3I). Setae *1* and *1a* on tergites IX and X of equal length (Fig. 3H). Dorsal lobe of Abd. XII with single median pore, ventral lobe with 1+1 *sal* pores. Female squama genitalis with distinct distal prolongation on stylus and long pointed acrostylus (Figs 3K, 5H). Male squama genitalis with 5+5 setae (Fig. 5I).

Body measurements (18 adults) (in µm): maximum body length 1150, head 135–138, pseudoculus 8–9, posterior part of maxillary gland 25–30; posterior cephalic setae *d7* 15–16, *sd7* 20–23, *l5* 7; pronotal setae *1* 35–48, *2* 12–16; mesonotal setae *P1* 35–45, *P2* 45–55; foretarsus 112–115, claw 28–30, empodial appendage 3.

#### Chaetal variability.

Asymmetrical absence of seta *A4* (5 specimens), and seta *A2* (2 specimens) on tergite VI, asymmetrical absence of seta *P1a* on tergite II (1 specimen), asymmetrical presence of additional small seta *P3a*’ on mesonotum (1 specimen, Fig. 2B).

#### Remarks.

This species belongs to the *confinis* group of *Acerentulus* species characterized by long foretarsal sensilla *a* and *b*; only *Acerentulus berruezanus* Aldaba, 1983 is characterized in possessing *P1a* setae on tergites I and VII (setae *P1a* absent on tergites II–VI). Other members of the *confinis* group are characterized by the absence of *P1a* setae on tergites I–VI. Three species from the *cunhai* group, *A. proximus* Szeptycki, 1997, *A. correseanus* Szeptycki, 1997 and *A. tuxeni* Rusek, 1966, have a similar chaetotaxy (setae *P1a* present on tergites II to VII, eight *A*-setae on tergite VII) and porotaxy (notal sterna and sternites I–III without pores, sternites IV–V with 1+1 *spsm* pores, sternite VII with a spm pore, sternite VI with two groups of *spsm* pores 2+2 or 2+3 in some males, except *A. tuxeni*, which has 1+1 *spsm* pores). However, as members of the *cunhai* group these three species have short sensilla *a* and *b*.

In preimago specimens the *A4* setae on tergite VII are absent symmetrically or asymmetrically. Setae *P1a* on tergites II–VII and *P3a* on tergite VII appear in the maturus junior stage (Fig. 4A, B). All pores on the tergites and sternites are present by the maturus junior stage, except for the posterosublateral pores (*psl*) on tergite VII, which appear in the preimago.

## Discussion

In a previous key to the *confinis* group (Shrubovych et al. 2012) the presence of six or eight anterior setae on tergite VI was used as the first character to divide the species. In the current study we found this character to be quite variable, with frequent asymmetrical absence of setae *A4* and *A2* on tergite VI. Szeptycki (1991) confirmed a high degree of variability of these characters in *Acerentulus exiguus* Condé, 1944, *A. xerophilus* Szeptycki, 1979, *A. cunhai* Condé, 1950, *A. traegardhi* Ionescu, 1937 and *A. tuxeni* Rusek, 1966. Therefore, it can be difficult to decide how many setae are present on tergite VI. Seta *A4* on tergite VI appears mostly in the adult stage (Aldaba 1984, Imadaté 1988), and this may be a reason for high variability of this character. Therefore, we have improved the identification key by using presumably more stable characters that appear in earlier stages of acerentomid postembryonic development, such as the presence of accessory setae *P1a* and *P3a* on tergites and seta on sternite XI in the maturus junior stage. According to Szeptycki (1991) sternal porotaxy is a good taxonomic character. Sternal pores are easily visible and practically identical with adult porotaxy from the maturus junior stage. Foretarsal sensillum shapes, proportional lengths, and location of sensilla and setae are also stable characters from larva II to adult (Shrubovych and Rusek 2010).

### Key to the *Acerentulus confinis* species group (valid from maturus junior stage)

**Table:** 

1	Tergite VII without *P3a* seta	**2**
–	Tergite VII with *P3a* seta	**7**
2	Sternite XI with 3+3 setae	**3**
–	Sternite XI with 2+2 setae	***A. halae* Szeptycki, 1997**
3	Foretarsal seta δ*4* in proximal position to base of *c*’	**4**
–	Foretarsal seta δ*4* in distal position to base of *c*’	***A. charrieri* Shrubovych, Schneider & D‘Haese, 2012**
4	Base of foretarsal sensillum *a*’ at level of seta *a3* insertion	**5**
–	Base of *a*’ distal to seta *a3* insertion, at level of seta δ*2* insertion	***A. occultus* Szeptycki, 1979**
5	Foretarsal sensillum *a* slender	**6**
–	Foretarsal sensillum *a* broadened basally	***A. apuliacus* Rusek & Stumpp, 1988**
6	Foretarsal sensillum *a*’ short, reaching to base of sensillum *t2*	***A. exiguus* Condé, 1944**
–	Foretarsal sensillum *a*’ long, reaching to base of sensillum *b*’	***A. carpaticus* Nosek, 1967**
7	Sternite XI with 3+3 setae	**8**
–	Sternite XI with 2+2 setae	***A. condei* Nosek, 1983**
8	Tergite VII without *Pla* seta	**9**
–	Tergite VII with *Pla* setae	**12**
9	Foretarsal sensillum *a* slender	**10**
–	Foretarsal sensillum *a* broad	***A. ochsenhausenus* Rusek, 1988**
10	Tergite VIII with 16 posterior setae (*P1* setae present)	**11**
–	Tergite VIII with 14 posterior setae (*P1* setae absent)	***A. alpinus* Gisin, 1945**
11	Foretarsal sensilla *c* and *b* equal in length, foretarsus long (110–125 µm)	***A. gisini* Condé, 1952**
–	Foretarsal sensillum *c* clearly longer than *b*, foretarsus shorter (about 80 µm)	***A. terricola* Rusek, 1965**
12	Foretarsal seta δ*4* in distal position to base of *c*’	**13**
–	Foretarsal seta δ*4* in proximal position to base of *c*’	**15**
13	Tergite VI with *P3a* setae	***A. gigas* Szeptycki, 1997**
–	Tergite VI without *P3a* seta	**14**
14	Tergite I with *Pla* seta, foretarsal sensilla *b* and *c* long, surpassing base of seta γ*3*	***A. berruezanus* Aldaba, 1983**
–	Tergite I without *Pla* seta, foretarsal sensilla *b* and *c* short, reaching to base of seta γ*3*	***A. confinis maderensis* Tuxen, 1982**
15	Tergites II–VI with *Pla* setae	***A. bulgaricus* sp. nov.**
–	Tergites II–VI without *Pla* seta	**16**
16	Foretarsal sensilla *b* and *c* equal in length	**17**
–	Foretarsal sensilla *b* and *c* differing in length	**20**
17	Foretarsal sensillum *a* long, surpassing base of seta γ*3*, foretarsus length about 80 µm	***A. setosus* Szeptycki, 1993**
–	Foretarsal sensillum *a* short, not reaching base of seta γ*3*, foretarsus longer than 100 µm	**18**
18	Foretarsal sensillum *c*’ long, surpassing base of claw, sternite VI with simple *spsm* pores, foretarsus length about 115 µm	***A. sinensis* Wu &Yin, 2007**
–	Foretarsal sensillum *c*’ short, not reaching base of claw, sternite VI with composed *spsm* pores	**19**
19	Sternite VII with seta *Pc*, foretarsus length about 100 µm	***A. xerophilus* Szeptycki, 1979**
–	Sternite VII without seta *Pc*, foretarsus length about 120 µm	***A. silvanus* Szeptycki, 1991**
20	Foretarsal sensillum *b* shorter than *c*	***A. palissai* Nosek, 1967**
–	Foretarsal sensillum *b* longer than *c*	**21**
21	Foretarsal sensillum *a* long, reaching to base of seta γ*3*; sensillum *d* long, surpassing base of *e*; length of foretarsus about 100 µm	***A. confinis* (Berlese, 1908)**
–	Foretarsal sensillum *a* short, not reaching to base of seta γ*3*; sensillum *d* short, not passing base of *e*; foretarsus length more than 110 µm	***A. alni* Szeptycki, 1991**

## Supplementary Material

XML Treatment for Acerentulus bulgaricus
